# The Normative Study of Acoustic and Aerodynamic Characteristics of Voice among Healthy Adult Turkish Speaker Population

**DOI:** 10.1155/2021/9217236

**Published:** 2021-12-15

**Authors:** Ayşegül Zencir Şen, Bülent Toğram

**Affiliations:** ^1^Kapadokya University, Faculty of Health Sciences, Department of Speech and Language Therapy, Nevşehir, Turkey; ^2^İzmir Bakırçay University, Faculty of Health Sciences, Department of Speech and Language Therapy, İzmir, Turkey

## Abstract

Phonatory Aerodynamic System (PAS Model 6600) is an evaluation instrument that assesses the effectiveness of surgical interventions, treatments, and therapy for voice disorders. It can be used for the assessment of voice disorders by supporting other perceptual and instrumental methods. It is important to establish normative data, because the use of appropriate norms is necessary for diagnostic and descriptive accuracy. Therefore, this study is aimed primarily at establishing adult normative databases for phonatory aerodynamic measures obtained with the KayPENTAX PAS Model 6600 among healthy adult Turkish speakers and then examining the effect of age, gender, and age-gender interaction variables on these measures. The contribution of the study is considered so important since it will generate normative data for all measurements—except the mean pitch—by the five protocols of PAS for the first time. Two hundred and six healthy Turkish speakers with normal voice (106 women and 100 men) were included in the study and stratified into three age groups. Forty-five phonatory aerodynamic measures across five PAS protocols (vital capacity, maximum sustained phonation, comfortable sustained phonation, variation in sound pressure level, and voicing efficiency) were collected. Age, gender, and age-gender interaction variables were analyzed for 45 PAS parameters. Significant gender and age effect was found for 30 and 19 variables, respectively. Gender-age interaction together was observed for only 6 parameters. Significant differences were not found for the remaining 10 parameters. Significant age and gender effects were observed for 35 phonatory and aerodynamic measures which are essential part of the objective clinical assessment of voice. Consequently, normative data used as reference in voice assessment should be generated according to age and gender differences.

## 1. Introduction

Healthy voice is of utmost importance for flawless communication. In some cases, communication might be interrupted by some voice disorders. The nature of voice disorders is complex, so is its assessment. For a comprehensive assessment of voice function, one needs to have a comprehensive story of the case, to mind the psychosocial context, and to evaluate the vibrational characteristics of the larynx and vocal folds. Among the means modern clinicians employ to evaluate voice are acoustic, aerodynamic, and endoscopic techniques—besides visual and perceptual assessment methods [1].

A comprehensive voice assessment constitutes a vital phase of an individualized and successful treatment program. The clinician should first conduct a comprehensive oral peripheral examination and a hearing test and should take a language and speech sample. The clinicians are advised to use all available perceptual and instrumental assessment methods they have [2].

In the evaluation of clients with voice disorders, whenever possible, the effect of the pathology on all relevant mechanisms/dimensions is determined by obtaining detailed information from the case about the voice complaint and following the following evaluation steps: auditory-perceptual evaluation, laryngeal endoscopic imaging, acoustics and aerodynamics evaluation, and the effect of voice disorder on their daily life [3, 4].

The acoustic analysis of voice function provides flawless noninvasive measurements of voice production, helps in discriminating normal from pathological voice, and measures the changes of voice in time [5]. Acoustic voice analysis helps to get information method in clinical research and useful in detecting underlying laryngeal pathologies with a close examination of signals emanating from the mouth in order to avoid misunderstanding that the voice analysis is the main part of the diagnostic process. Moreover, acoustic measurements are a common and significant means to evaluate the efficiency of voice therapy practices [6].

Aerodynamic measurements indicate the measurements of the flow, volume, and average pressure of the air produced by respiratory, laryngeal, and supralaryngeal airway mechanisms. Experts use aerodynamic measurements to evaluate dysphonia, to monitor voice changes during treatment, and to distinguish laryngeal and respiratory problems from each other [7].

Since human voice has a complex structure and influenced by individual characteristics, the definition of standard normal voice is so important. Voice disorders can be seen in all ages and in all genders; thus, normative values of voice for all age groups should be determined for objective assessment [8].

Phonatory Aerodynamic System (PAS Model 6600) (KayPENTAX Corp., Lincoln Park, NJ) is an instrument used in the assessment of voice disorders. It also enables the researchers to get objective measurements about the acoustic and aerodynamic characteristics of voice. PAS is an evaluation instrument that assesses the effectiveness of surgical interventions, treatments, and therapy for voice disorders. It can be used for the assessment of voice disorders by supporting other perceptual and instrumental methods. PAS consists of seven protocols: (1) air pressure screening (APSC), (2) vital capacity (VC), (3) maximum sustained phonation (MSPH), (4) comfortable sustained phonation (CSPH), (5) variation in sound pressure level (VSPL), (6) voicing efficiency (VOEF), and (7) running speech (RS). By these protocols, 45 acoustic and aerodynamic measurements can be collected.

First known report of adult normative data obtained with PAS 6600 was published in 2013 by Zraick et al. [9] A sample of 89 female and 68 male participants (157 participants per total) took part in the research, and they were divided into three age groups (18-39, 40-59, and 60+ years). To establish normative data, six PAS protocols were applied to participants. Researchers obtained normative data for 45 acoustic and aerodynamic parameters and investigated whether the data obtained age and gender dependent. In their study, Weinrich et al. aimed to (1) establish normative data of PAS measurements in the pediatric groups and to (2) determine whether age and/or gender has an influence on the outcomes [10]. Finally, Kim reported adult normative data for some phonatory aerodynamic parameters in Korea. In this research, Kim examined 70 male and 100 female participants between the ages of 18 and 49 years. Maximum sustained phonation and voicing efficiency protocols of PAS were applied to participants. Normative data for 25 acoustic and aerodynamic measurements were obtained, and intrasubject reliability was tested. In this study, in contrast to the former two, the age variable was excluded, and only the influence of gender was examined [11].

The primary purpose of the present study is to establish adult normative data for 45 acoustic and aerodynamic parameters obtained with PAS Model 6600 in Turkish-speaking healthy adults without voice disorders, considering the variables of age, gender, and gender-age interaction. In the related literature in Turkish, normative data were only gathered for fundamental frequency and perturbation measurements. Therefore, contribution of the present study is considered so important since it will generate normative data for all measurements—except the mean pitch—by the five protocols of PAS for the very first time.

The main questions of the study are as follows:

1. Are the acoustic and aerodynamic measurements obtained from PAS protocols influenced by gender, age, and gender-age interaction variables?
2. What are the norm values of PAS for Turkish-speaking healthy adults?

## 2. Materials and Methods

### 2.1. Design

In the present study, normative data for acoustic and aerodynamic parameters of PAS are established by intergroup prospective data acquisition pattern method. The independent variables of the research are age and gender. The dependent variables are 45 phonatory measurements in the five protocols of PAS.

### 2.2. Participants

Normative data was collected from 206 adults. The ages of the 206 participants (106 females and 100 males) of this research vary from 18 to 87. The participants' mean ages were 46 and 83 (SD = 17, 45). Female participants' mean ages are 46 and 64 (SD = 17, 51), and male participants' mean ages are 47 and 67 (SD = 17, 44). The participants are categorized in three age groups (18-39, 40-59, and 60+), and each age group is categorized into two according to gender. Thus, the total number of groups is six. In the 18–39 age group, there are 41 female participants (mean ages 28, 27 (SD = 7, 07)) and 35 male participants (mean ages 28, 37 (SD = 6, 56)). In the 40–59 age group, there are 35 female participants (mean ages 47, 63 (SD = 5, 70)) and 35 male participants (mean ages 48, 86 (SD = 5, 61)). In the 60+ age group, there are 30 female participants (mean ages 68, 47 (SD = 5, 77)) and 30 male participants (mean ages 68, 80 (SD = 6, 21)).

The participants of the study were monolingual Turkish-speaking volunteer adults who do not have any breathing problems, any hearing and speaking disorders, any neurological problems, and who do not smoke. All participants were evaluated informally and perceptually in terms of language, speech, and voice during the clinical interview by two speech and language therapists who are experts in the field of speech and language therapy. Moreover, the participants were required to evaluate their own voices and to fill in Voice Handicap Index. Participants who had voice disorders were not included in the research. On the day of data collection, participants who had flu or seasonal allergies and female participants in their menstruation period were also excluded.

### 2.3. Instrumentation

In this study, PAS Model 6600 was used to obtain acoustic and aerodynamic data from the participants. The PAS is a computer-based software and hardware system designed to measure airflow, air pressure, and other acoustic and aerodynamic characteristics about voice, and it includes measurements of mean phonatory flow velocity, sound pressure level, base frequency, vital capacity, subglottal resistance and subglottal pressure, and efficiency measurements. PAS is able to record and monitor phonatory aerodynamic data simultaneously [5]. PAS consists of seven protocols: air pressure screening, vital capacity, maximum sustained phonation, comfortable sustained phonation, variation in sound pressure level, voicing efficiency, and running speech. These protocols provide 45 acoustic and aerodynamic measurements. With the data acquired by PAS, 29 statistics can be calculated, and the wave for each of the sound can be sampled and played on the computer card. The hardware of the portable PAS device consists of an integrated microphone, an airflow cap, a mask and an airflow tube, and an intraoral tube. The software uses Microsoft Windows 2000/XP.

### 2.4. Procedure

This study was approved by the Ethical Committee of Anadolu University. Participant notification and consent forms were presented to the attention of all participants; they were informed about the research, their rights, responsibilities, and the researchers' responsibilities in detail. The participants who read the consent form and enrolled the research were also required to fill in the participant information form which was developed by the researches and which included demographic data such as name, surname, age, educational background, voice health story, and health story of the participants.

All data collection in this study was completed in the phoniatry unit of The Speech and Language Disorders Education and Research Center in Anadolu University. Each participant was seated in a sound-treated room and only the participant and the first author of the research occurred in the room at the time of PAS procedure. Researchers demonstrated each PAS protocol to each participant, and then, each session was conducted.

45 acoustic and aerodynamic measurements were acquired throughout the six PAS protocols. The following issues were specially paid attention at the beginning of each session: The device was calibrated according to the manufacturer's instructions before every session, and the mask was cleaned with antiseptic cleaners before and after each session. The mask always covered the face of each participant in a certain way that did not lose air, and each participant was encouraged to speak in their most comfortable vocal pitch and loudness. The participants practiced the tasks standing or sitting, as they wished, and the tasks were repeated when necessary. The average duration for data acquisition was 20 minutes. After each participant went through the air pressure screening protocol, the following five PAS protocols were practiced three times each: vital capacity, maximum sustained phonation, comfortable sustained phonation, variation in sound pressure level, and voicing efficiency. The average measurements were recorded for statistical analysis. Each PAS protocol has its own automatic threshold algorithms, which were used for the analysis of the results. When necessary, the threshold algorithms were manipulated manually according to PAS manual (KayPENTAX, Lincoln Park, NJ). The PAS protocols conducted are as follows:

- Air pressure screening (APSC): The main aim of this protocol was screening. Intraoral tube and leak tube were used together in this protocol. Intraoral tube was positioned between the lips and the leak tube on the corner of the two lips. The participant was required to apply a pressure of 5 cm H_2_O for 5 seconds. The participants who passed this protocol were required to continue with the following
- Vital capacity (VC): The participant, while the mask was on, was required to take a deep breath and exhale into the mask as if she was exhaling towards a candle at a distance until she was run out of breath. The measurements obtained from this protocol included expiratory airflow duration, peak expiratory airflow, and expiratory volume
- Maximum sustained phonation (MSPH): The participant was required to take a deep breath with the mask on and to sustain the phonation /ʌ:h/ in natural pitch and volume as long as she can. The measurements obtained from this protocol included maximum SPL, minimum SPL, mean SPL, SPL range, mean SPL during voicing, mean pitch, phonation time, peak expiratory airflow, mean expiratory airflow, and expiratory volume
- Comfortable sustained phonation (CSPH): The participant was required to take a deep breath with the mask on and to sustain the phonation /ʌ:h/ in natural pitch and volume for seven seconds. The first and the last seconds were excluded from the analysis, and five seconds in between was analyzed. The measurements obtained from this protocol included maximum SPL, minimum SPL, mean SPL, SPL range, mean pitch, phonation time, peak expiratory airflow, mean expiratory airflow, and expiratory volume
- Variation in sound pressure level (VSPL): The participant, with the mask on, was required to repeat the syllable /pa:pa:pa/ three times without any phonation break in three different loudness levels (comfortable, soft, and loud). The measurements obtained from this protocol included maximum SPL, minimum SPL, mean SPL, SPL range, mean pitch, pitch range, and target airflow
- Voicing efficiency (VOEF): The participant was required to repeat the syllable /apapapa/ seven times in their comfortable loudness level and without taking a breath in between. The first and the last syllables were excluded, and five syllables in between were analyzed. The measurements obtained from this protocol included maximum SPL, mean SPL, mean SPL during voicing, mean pitch, pitch range, expiratory airflow duration, peak air pressure, mean peak air pressure, peak expiratory airflow, target airflow, expiratory volume, mean airflow during voicing, aerodynamic power, aerodynamic resistance, aerodynamic efficiency, and acoustic ohms

### 2.5. Statistical Analysis

IBM SPSS Statistics 21 and Sigma Stat 3.5 were used for data analysis. Data were transferred to Sigma Stat 3.5 software and went through to two-way analysis of variance. In the cases where a significant difference was detected among groups, Holm-Sidak test was employed to determine which group was the source of difference.

## 3. Results

In this research, normative data for 45 acoustic and aerodynamic measurements of PAS were acquired, and the influence of age, gender, and gender-age interaction was analyzed. The findings indicated that age variables influenced 19 parameters, gender variables influenced 30 parameters, and gender-age interaction influenced 6 parameters. The remaining 10 parameters did not reach statistical significance. *P* values of PAS parameters, intergroup comparison findings, and data on power analysis can be found in Table 1. ANOVA results of all parameters of PAS protocol can be found in Table 2.

For the second aim of the study, the acquired data was analyzed according to the variables of age, gender, and gender-age interaction, and the normative data for the acoustic and aerodynamic PAS parameters were established.  Table 3 includes the data on the means, standard deviations, and range of normative data obtained from 157 participants. And this data is categorized into age and gender groups for all parameters of all PAS protocols.

## 4. Discussion

The main aim of this study is to establish normative data for the acoustic and aerodynamic characteristics of voice in Turkish-speaking healthy adults, by using PAS protocols. The study examines the influence of the variables of age, gender, and gender-age interaction. The present study is the third research in the literature after the researches of Zraick et al. [9] and Kim [11]. The findings of the present study were initially compared with the findings of these two because such a comparison with other studies is limited since different tools were used in each study, and their age groups were different from each other. However, other studies were cited when possible.

In this section, the comparison among these studies primarily demonstrated the influence of the variables of age, gender, and gender-age interaction on the parameters. Then, for the second aim of the study, the parameters of PAS protocol were categorized in groups such as *airflow measurement*, *sound pressure level measurement*, *pitch measurement*, and *air pressure measurement*. The influences of the variables age, gender, and gender-age interaction on these measurements were compared to the findings of other studies.

### 4.1. Age Effects

Age variable has effects on expiratory volume in vital capacity protocol; on maximum SPL, minimum SPL, SPL range, phonation time, peak expiratory airflow, and expiratory volume in maximum sustained phonation protocol; on mean SPL, pitch range, and target airflow in variation in sound pressure level protocol; and on maximum SPL, mean SPL, expiratory airflow duration, peak air pressure, mean peak air pressure, expiratory volume, aerodynamic resistance, and acoustic ohm measurements in voicing efficiency protocol.

### 4.2. Gender Effects

Gender variable has effects on expiratory volume, expiratory airflow duration, and peak expiratory airflow in vital capacity protocol; on mean pitch, phonation time, peak expiratory airflow, mean expiratory airflow, and expiratory volume in maximum sustained phonation protocol; on mean pitch, peak expiratory airflow, mean expiratory airflow, and expiratory volume in comfortable sustained phonation protocol; on minimum SPL, mean SPL, mean pitch, pitch range, and target airflow in variation in sound pressure level protocol; and on mean pitch, pitch range, expiratory airflow duration, peak air pressure, mean peak air pressure, peak expiratory airflow, target airflow, expiratory volume, mean airflow during voicing, aerodynamic power, aerodynamic resistance, acoustic ohms, and aerodynamic efficiency measurements in voicing efficiency protocol.

### 4.3. Age and Gender Interaction

Age-gender interaction variable has effects on minimum SPL, SPL range, and mean pitch in maximum sustained phonation protocol; on mean pitch in comfortable sustained phonation protocol; on mean pitch in variation in sound pressure level protocol; and on mean pitch measurements in voicing efficiency protocol.

The changes in voice resulting from ageing consist of pitch changes, irregular vocal fold vibrations, glottal inefficacies, air loss and breathy voice, laryngeal tension, and muffled voice. As Kahane [12] claims, the indications of ageing are more obvious in male voices; however, both male and female voices age. The findings of perceptual, physiologic, and acoustic studies show that the changes in voice resulting from ageing depend on the transformation of larynx tissues and on anatomic and physiologic transformations. In our study, the findings of the measurements related to the airflow, i.e., *expiratory volume*, *peak expiratory airflow*, *mean expiratory airflow during voicing*, *target airflow*, and *mean air flow during voicing*, show that anatomical and physiological transformation in the laryngeal mechanism due to ageing affects airflow: Airflow measurements decrease due to ageing. Related literature includes many studies that show ageing has different effects on the phonatory behaviors of women and men. Our findings are in accord with the findings of these studies [9, 13–17]. The shared finding about age and gender is that the effects of ageing are much more obvious on male voices than on female voices. As for the gender variable in airflow measurements, again in consistency with our findings, the literature shows that airflow measurements of men are significantly higher than those of women in all age groups [9, 11, 14, 18, 19]. This is claimed to be the result of anatomic, physiologic, and structural differences in their respiratory and laryngeal systems.

Our findings in *mean pitch* and *pitch range* measurements are also consistent with the findings in the related literature [9, 11, 20]. In all age groups, the mean pitch of female voice is higher than that of male voice. However, the pitch of the female voice decreases with age, while that of the male voice increases.

Our findings in sound pressure level measurements, such as *maximum SPL*, *minimum SPL*, *mean SPL during voicing*, and *SPL range*, are all higher in all age and gender groups than the findings in the studies of Zraick et al. [9] and Kim [11]. This is considered the result of the stressed structure of Turkish language. According to our findings, sound pressure levels increase with age. Although there is no statistically significant difference between age groups, the correlations between age and sound pressure levels in our findings are in consistency with those of Zraick et al. [9]. The pressure on laryngeal muscles increases with age, and the participants have to make more effort to start phonation; therefore, the sound pressure levels increase.

In air pressure measurements, significant differences between age and gender groups are observable. These findings are consistent with the findings of Higgins and Saxman [14], in which *subglottal pressures* of male participants over 69 years of age are significantly higher than those of young males from 20 to 31 years of age. Age variable is expected to affect *subglottal pressure* values, such as vocal fold tension and incomplete glottal closure [13]. The significant differences in our findings between gender groups are consistent with Kim [11], in which the measurements of male participants are higher than those of females.

For the second purpose of the study, the 157 participants were categorized into three age groups (18-39, 40-59, and 60+), and the normative data for all acoustic and aerodynamic measurements in PAS were determined. In Kim's study (2014), there is only one age group (18-49), and the norms are determined only for the acoustic and aerodynamic measurements in the two protocols, i.e., maximum sustained phonation and voicing efficiency. Therefore, the findings of the present study can only be compared with those of Zraick et al. [9]. In both studies, the normative data and standard deviations of airflow measurements such as *expiratory airflow duration*, *maximum expiratory airflow*, *expiratory volume*, *mean expiratory airflow*, *target airflow*, *expiratory airflow duration*, *mean airflow duration during voicing*, and *phonation duration* are expectedly similar. However, the normative data and standard deviations in sound pressure level measurements of PAS protocols, such as *maximum sound pressure level*, *minimum sound pressure level*, *mean sound pressure level*, *sound pressure level interval*, and *mean sound pressure during voicing*, are higher in our findings than in those of Zraick et al. [9] in all age and gender groups. This difference is considered the result of the stressed structure of Turkish language. Norms and standard deviations in pitch measurements of PAS protocols, such as mean *pitch* and *pitch range*, are also higher in our findings. These findings indicate that healthy Turkish-speaking adults use their voice in higher pitches. Similarly, the norms for measurements of air pressure in the voicing efficiency protocol are higher than those of Zraick et al.'s study [9]. This difference is considered the result of the language used by participants and their efforts to start voicing.

Finally, the subjects of the study have not been endoscopically examined because they have had no breathing or voice problem on the day of data collection. Moreover, subjects were perceptually evaluated by two experienced clinicians with expertise in speech and language therapy. This is considered as the limitation of the study, and laryngeal examination is recommended in similar studies.

The findings of the present study show that acoustic and aerodynamic measurements are responsive to age and gender differences, and these findings are consistent with those of other studies in the related literature [9, 11, 17]. Further research may be designed for norm generation using PAS with a wider group of participants. The age groups may be narrowed, so that the number of age groups may increase.

## 5. Conclusions

In this study, our findings indicate that acoustic and aerodynamic measurements, which are so crucial in voice assessment, are so sensitive to age and gender differences. Therefore, normative data used as reference in voice assessment should be generated according to age and gender differences.

## Figures and Tables

**Table 1:** ANOVA results for each parameter within each protocol.

Protocol and parameter	*P* value	Post hoc results	Power
Vital capacity			
Expiratory airflow duration			
Gender	<0.001	Males > females	0.942 (>80)
Peak expiratory airflow			
Gender	<0.001	Males > females	0.988 (>80)
Expiratory volume			
Age	<0.001		1.000 (>80)
	<0.05	Younger adults > older adults	
	<0.05	Middle-aged adults > older adults	
	<0.05	Younger adults > middle-aged adults	
Gender	<0.001	Males > females	1.000 (>80)
Maximum sustained phonation			
Maximum SPL			
Age	<0.05	Middle-aged adults > younger adults	0.621 (>60)
Minimum SPL			
Age	<0.01	Younger adults > older adults	0.621 (>60)
	<0.05		
Gender-age 18-39 age	<0.05		0.627 (>60)
Females	<0.05	Females > males	
	<0.05		
Erkek	NS	Younger adults > older adults Middle-aged adults > older adults	
SPL range			
Age	<0.001	Older adults > younger adults Middle-aged adults > younger adults	0.924 (>80)
	<0.05		
	<0.05		
Gender-age 18-39 age 40-59 age	<0.01	Males > females Males > females	0.619 (>60)
	<0.05		
	<0.05		
Females	<0.05	Older adults > younger adults	
	<0.05	Older adults > middle-aged adults	
	<0.05	Middle-aged adults > younger adults	
Males	NS		
Mean pitch			
Gender	<0.001	Females > males	1.000 (>80)
Gender-age	<0.001		0.996 (>80)
18-39 age	<0.05	Females > males	
40-59 age	<0.05	Females > males	
60+ age	<0.05	Females > males	
Females	<0.05	Younger adults > older adults	
	<0.05	Middle-aged adults > older adults	
Males	<0.05	Older adults > younger adults	
	<0.05	Middle-aged adults > younger adults	
Phonation time			
Age	<0.001	Younger adults > older adults Younger adults > middle-aged adults	0.996 (>80)
	<0.05		
	<0.05		
Gender	<0.001	Males > females	0.996 (>80)
Peak expiratory airflow			
Age	<0.05	Middle-aged adults > younger adults	0.518
Gender	<0.001	Males > females	0.919 (>80)
Mean expiratory airflow			
Gender	<0.001	Males > females	1.000 (>80)
Expiratory volume			
Age	<0.001	Younger adults > older adults Middle-aged adults > older adults	0.986 (>80)
	<0.05		
	<0.05		
Gender	<0.001	Males > females	1.000 (>80)
Comfortable sustained phonation			
Mean pitch			
Gender	<0.001	Females > males	1.000 (>80)
Gender-age	<0.001		
18-39 age	<0.05	Females > males	0.983 (>80)
40-59 age	<0.05	Females > males	
60+ age	<0.05	Females > males	
Females	<0.05	Younger adults > older adults	
	<0.05	Middle-aged adults > older adults	
Males	<0.05	Older adults > younger adults	
	<0.05	Middle-aged adults > younger adults	
Peak expiratory airflow			
Gender	<0.01	Males > females	0.698 (>60)
Mean expiratory airflow			
Gender	<0.001	Males > females	1.000 (>80)
Expiratory volume			
Gender	<0.001	Males > females	1.000 (>80)
Variation in SPL			
Minimum SPL			
Gender	<0.05	Males > females	0.621 (>60)
Mean SPL			
Gender	<0.001	Males > females	0.621 (>60)
Age	<0.001	Older adults > younger adults	0.921 (>80)
	<0.001	Middle-aged adults > younger adults	
Mean pitch			
Gender	<0.001	Females > males	1.000 (>80)
Gender-age	<0.001		1.000 (>80)
18-39 age	<0.05	Females > males	
40-59 age	<0.05	Females > males	
60+ age	<0.05	Females > males	
Females	<0.05	Younger adults > older adults	
Males	<0.05	Older adults > younger adults	
	<0.05	Older adults > middle-aged adults	
Pitch range			
Age	<0.05	Middle-aged adults > younger adults	0.619 (>60)
Gender	<0.001	Females > males	0.993 (>80)
Target airflow			
Age	<0.05	Middle-aged adults > older adults	0.479
Gender	<0.001	Males > females	1.000 (>80)
Voicing efficiency			
Maximum SPL			
Age	<0.05	Middle-aged adults > younger adults	0.631 (>60)
Mean SPL			
Age	<0.05	Middle-aged adults > younger adults	0.609 (>60)
Mean pitch			
Gender	<0.001	Females > males	1.000 (>80)
Gender-age	<0.001		1.000 (>80)
Females	<0.001	Younger adults > older adults	
	<0.001	Middle-aged adults > older adults	
Males	<0.001	Older adults > younger adults	
	<0.05	Middle-aged adults > younger adults	
Pitch range			
Gender	<0.001	Females > males	1.000 (>80)
Expiratory airflow duration			
Age	<0.001	Older adults > younger adults Middle-aged adults > younger adults	0.998 (>80)
	<0.05		
	<0.05		
Gender	<0.05	Females > males	0.383
Peak air pressure			
Age	<0.01	Older adults > younger adults	0.786 (>60)
Gender	<0.001	Males > females	0.909 (>80)
Mean peak air pressure			
Age	<0.01	Older adults > younger adults	0.684 (>60)
Gender	<0.01	Males > females	0.786 (>60)
Peak expiratory airflow			
Gender	<0.001	Males > females	1.000 (>80)
Target airflow			
Gender	<0.001	Males > females	1.000 (>80)
Expiratory airflow			
Age	<0.01	Middle-aged adults > younger adults	0.772 (>60).
Gender	<0.001	Males > females	1.000 (>80)
Mean airflow during voicing			
Gender	<0.001	Males > females	1.000 (>80)
Aerodynamic power			
Gender	<0.001	Males > females	1.000 (>80)
Aerodynamic resistance			
Age	<0.001	Older adults > middle-aged adults Older adults > younger adults	0.892 (>80)
	<0.05		
	<0.05		
Gender	<0.05	Females > males	0.379
Acoustic ohms			
Age	<0.001	Older adults > middle-aged adults Older adults > younger adults	0.894 (>80)
	<0.05		
	<0.05		
Gender	<0.05	Females > males	0.383
Aerodynamic efficiency			
Gender	<0.01	Females > males	0.671 (>60)

**(a):** 

Vital capacity			
Source	*F*	*P*	Power
Expiratory airflow duration			
Age	1.061	0.348	0.0575
Gender	12.595	<0.001^∗∗∗^	0.942
Gender-age	0.523	0.594	0.0500
Peak expiratory airflow			
Age	2.569	0.079	0.318
Gender	15.794	<0.001^∗∗∗^	0.988
Gender-age	1.149	0.542	0.0500
Expiratory volume			
Age	27.081	<0.001^∗∗∗^	1.000
Gender	111.939	<0.001^∗∗∗^	1.000
Gender-age	1.867	0.157	0.189

^∗∗∗^Sidak < 0.001.

**(b):** 

Maximum sustained phonation						
Source	*F*	*P*	Power	*F*	*P*	Power
Maximum SPL				Phonation time		
Age	4.324	0.015^∗^	0.621	12.319	<0.001^∗∗∗^	0.996
Gender	0.535	0.535	0.0500	19.458	<0.001^∗∗∗^	0.996
Gender-age	0.770	0.770	0.0500	1.863	0.158	0.188

Minimum SPL				Peak expiratory airflow		
Age	4.402	0.08^∗∗^	0.632	3.678	0.027^∗^	0.518
Gender	1.088	0.298	0.0568	11.623	<0.001^∗∗∗^	0.919
Gender-age	4.367	0.014^∗^	0.014	0.102	0.903	0.0500

Mean SPL				Mean expiratory airflow		
Age	1.830	0.163	0.183	2.565	0.079	0.317
Gender	1.500	0.222	0.101	34.733	<0.001^∗∗∗^	1.000
Gender-age	0.833	0.436	0.0500	0.310	0.734	0.0500

SPL range				Expiratory volume		
Age	7.662	<0.001^∗∗∗^	0.924	10.393	<0.001^∗∗∗^	0.986
Gender	1.634	0.203	0.116	89.935	<0.001^∗∗∗^	1.000
Gender-age	4.127	0.018^∗^	0.591	2.124	0.122	0.235

Mean SPL during voicing				Mean pitch		
Age	2.342	0.099	0.275	1.869	0.157	0.189
Gender	0.015	0.747	0.0500	321.984	<0.001^∗∗∗^	1.000
Gender-age	1.236	0.293	0.0839	12.307	<0.001^∗∗∗^	0.996

^∗^Sidak < 0.05. ^∗∗∗^Sidak < 0.001.

**(c):** 

Comfortable sustained phonation						
Source	*F*	*P*	Power	*F*	*P*	Power
Maximum SPL				Phonation time		
Age	1.485	0.229	0.124	1.125	0.327	0.0670
Gender	0.314	0.576	0.0500	0.00679	0.934	0.0500
Gender-age	0.522	0.594	0.0500	0.339	0.713	0.0500

Minimum SPL				Peak expiratory airflow		
Age	0.572	0.566	0.0500	0.829	0.438	0.0501
Gender	1.138	0.287	0.0621	7.121	0.008^∗∗^	0.0520
Gender-age	0.695	0.500	0.0500	0.652	0.522	0.0562

Mean SPL				Mean expiratory airflow		
Age	1.830	0.163	0.183	1.176	0.311	0.747
Gender	1.500	0.222	0.101	27.823	<0.001^∗∗∗^	1.000
Gender-age	0.833	0.436	0.0500	0.0721	0.930	0.0500

SPL range				Expiratory volume		
Age	1.637	0.197	0.149	1.198	0.304	0.0781
Gender	1.291	0.257	0.0785	27.808	<0.001^∗∗∗^	1.000
Gender-age	0.0912	0.913	0.0500	0.0905	0.913	0.0500

Mean pitch						
Age	1.345	0.263	0.101			
Gender	322.828	<0.001^∗∗∗^	1.000			
Gender-age	10.108	<0.001^∗∗∗^	0.983			

^∗∗^Sidak < 0.01. ^∗∗∗^Sidak < 0.001.

**(d):** 

Variation in sound pressure level						
Source	*F*	*P*	Power	*F*	*P*	Power
Maximum SPL				Mean pitch		
Age	1.833	0.163	0.183	0.211	0.810	0.0500
Gender	0.562	0.454	0.0500	408.017	<0.001^∗∗∗^	1.000
Gender-age	1.170	0.313	0.0738	15.688	<0.001^∗∗∗^	1.000

Minimum SPL				Pitch range		
Age	3.298	0.039^∗^	0.452	4.314	0.015^∗^	0.619
Gender	6.203	0.014^∗^	0.621	18.349	<0.001^∗∗∗^	0.993
Gender-age	1.539	0.217	0.133	1.631	0.198	0.148

Mean SPL				Target airflow		
Age	1.830	0.163	0.183	3.456	0.033^∗^	0.479
Gender	1.500	0.222	0.101	95.265	<0.001^∗∗∗^	1.000
Gender-age	0.833	0.436	0.0500	0.0605	0.941	0.0500

SPL range						
Age	2.473	0.087	0.300			
Gender	2.865	0.092	0.258			
Gender-age	0.944	0.391	0.0500			

^∗^Sidak < 0.05. ^∗∗∗^Sidak < 0.001.

**(e):** 

Voicing efficiency						
Source	*F*	*P*	Power	*F*	*P*	Power
Maximum SPL				Peak expiratory airflow		
Age	4.394	0.014^∗^	0.631	1.121	0.328	0.0665
Gender	0.200	0.655	0.0500	76.064	<0.001^∗∗∗^	1.000
Gender-age	0.236	0.790	0.0500	0.777	0.461	0.0500

Mean SPL				Target airflow		
Age	4.248	0.016^∗^	0.609	1.929	0.148	0.200
Gender	0.386	0.535	0.0500	70.980	<0.001^∗∗∗^	1.000
Gender-age	0.143	0.867	0.0500	0.284	0.753	0.0500

Mean SPL during voicing				Expiratory volume		
Age	2.893	0.058	0.378	5.509	0.005^∗∗^	0.772
Gender	0.223	0.223	0.100	37.879	<0.001^∗∗∗^	1.000
Gender-age	0.538	0.538	0.0500	0.706	0.495	0.0500

Mean pitch				Mean airflow during voicing		
Age	1.071	0.345	0.0590	1.838	0.162	0.184
Gender	3.730	0.055	0.360	68.253	<0.001^∗∗∗^	1.000
Gender-age	0.364	0.364	0.0511	0.227	0.797	0.0500

Pitch range				Aerodynamic power		
Age	0.632	0.532	0.0500	0.504	0.605	0.0500
Gender	25.499	<0.001^∗∗∗^	1.000	55.450	<0.001^∗∗∗^	1.000
Gender-age	0.249	0.780	0.0500	0.0126	0.987	0.0500

Expiratory airflow duration				Aerodynamic resistance		
Age	13.205	<0.001^∗∗∗^	0.998	7.006	<0.001^∗∗∗^	0.892
Gender	3.928	0.049^∗^	0.383	3.899	0.050	0.379
Gender-age	0.635	0.531	0.0500	1.425	0.243	0.114

Peak air pressure				Aerodynamic efficiency		
Age	5.654	0.004^∗∗^	0.786	1.728	0.180	0.165
Gender	11.254	<0.001^∗∗∗^	0.909	6.791	0.01^∗∗^	0.671
Gender-age	1.619	0.201	0.146	1.691	0.187	0.158

Mean peak air pressure				Acoustic ohms		
Age	4.770	0.009^∗∗^	0.684	7.053	<0.001^∗∗∗^	0.894
Gender	8.407	0.004^∗∗^	0.786	3.928	0.049^∗∗^	0.383
Gender-age	0.662	0.517	0.0500	1.447	0.238	0.117

^∗^Sidak < 0.05. ^∗∗^Sidak < 0.01. ^∗∗∗^Sidak < 0.001.

**Table:** (a) Vital capacity

Protocol parameter		18–39 y (*n* = 76)		40–59 y (*n* = 70)	60–87 y (*n* = 60)	
Vital capacity	Mean (SD)	Min–Max	Mean (SD)	Min–Max	Mean (SD)	Min–Max
Female	*n* = 41		*n* = 35		*n* = 30	
Expiratory airflow duration (s)	6.15(2.39)	2.41-13.57	6.42 (2.88)	2.40-14.26	5.57 (1.83)	2.07-9.31
Peak expiratory airflow (L/s)	0.94 (0.62)	0.27-3.18	1.16 (0.96)	0.29-5.15	1.18 (0.73)	0.30-3.78
Expiratory volume (L)	2.42 (0.63)	1.03-3.58	2.21 (0.61)	0.91-3.66	1.70 (0.55)	0.86-2.92

Male	*n* = 35		*n* = 35		*n* = 30	
Expiratory airflow duration (s)	7.91 (3.21)	3.22-14.73	7.27 (3.09)	2.40-14.71	7.12 (1.65)	1.65-13.50
Peak expiratory airflow (L/s)	1.43 (1.02)	0.17-5.19	1.93 (1.35)	0.28-6.71	1.59 (0.91)	0.28-3.42
Expiratory volume (L)	3.83 (1)	1.63-6.04	3.35 (0.86)	1.05-5.20	2.59 (0.87)	0.68-4.11

**Table:** (b) Maximum sustained phonation

Protocol parameter		18–39 y (*n* = 76)		40–59 y (*n* = 70)	60–87 y (*n* = 60)	
Maximum sustained phonation	Mean (SD)	Min–Max	Mean (SD)	Min–Max	Mean (SD)	Min–Max
Female	*n* = 41		*n* = 35		*n* = 30	
Maximum SPL (dB)	99.50 (4.98)	91.68–112.26	101.17 (5.33)	93.43-112.71	100.78 (4.23)	94.09-110.36
Minimum SPL (dB)	68.05 (12.56)	44.85-88.43	63.86 (11.96)	46.78-92.70	56.18 (11.77)	42.58-85.43
Mean SPL (dB)	94.47 (5.02)	86.82-105.39	95.35 (5.20)	87.66-105.86	94.06 (4.76)	84.78-103.17
SPL range (dB)	31.44 (12.68)	6.4-60.7	37.25 (12.31)	12.94-61.53	44.64 (12.69)	12.46-63.43
Mean SPL during voicing (dB)	94.75 (4.99)	86.91-106.28	95.34 (7.43)	64.75-108.42	95.09 (4.63)	85.44-103.56
Mean pitch (Hz)	222.47 (22.33)	161.99-260.87	222.64 (27.76)	155.93-257.32	202.39 (29.15)	142.21-245.33
Phonation time (s)	17.53 (4.87)	6.88-29.56	16.51 (5.52)	7.45-27.64	14.26 (4.23)	8.58-23.03
Peak expiratory airflow (L/s)	0.21 (0.10)	0.03-0.6	0.34 (0.22)	0.08-1.01	0.25 (0.11)	0.01-0.47
Mean expiratory airflow (L/s)	0.10 (0.04)	0.02-0.2	0.11 (0.04)	0.05-0.23	0.10 (0.05)	0.0067-0.21
Expiratory volume (L)	1.77 (0.77)	0.31-3.31	1.81 (0.59)	0.59-2.89	1.37 (0.56)	0.06-2.28

Male	*n* = 35		*n* = 35		*n* = 30	
Maximum SPL (dB)	99.28 (3.25)	91.89-105.47	102.05 (5.44)	91.94-112.37	101.35 (4.62)	90.59-107.38
Minimum SPL (dB)	62.18 (14.04)	43.09-90.14	58.80 (12.17)	42.98-83.55	61.76 (10.09)	46.02-81.35
Mean SPL (dB)	93.53 (3.27)	84.17-101.32	96.55 (5.46)	85.01-108.18	94.14 (7.60)	74.32-103.92
SPL range (dB)	37.13 (14.66)	8.44-59.42	43.24 (11.48)	11.31-61.49	39.59 (9.10)	20.85-57.46
Mean SPL during voicing (dB)	93.93 (3.30)	84.63-101.49	97.30 (5.31)	86.71-108.47	94.72 (7.19)	77.51-103.99
Mean pitch (Hz)	131.19 (18.67)	100.81-171.67	148.74 (27.90)	107.42-210.70	158.48 (39.17)	97.52-255.04
Phonation time (Sn)	22.75 (5.07)	11.59-29.58	18.45 (6.19)	6.29-29.74	17.00 (5.89)	6.11-29.44
Peak expiratory airflow (L/s)	0.34 (0.15)	0.08-0.95	0.49 (0.52)	0.06-2.76	0.43 (0.50)	0.05-2.98
Mean expiratory airflow (L/s)	0.15 (0.05)	0.04-0.26	0.16 (0.07)	0.02-0.32	0.13 (0.06)	0.01-0.26
Expiratory volume (L)	3.28 (1.04)	0.97-5.08	2.84 (1.07)	0.50-5.03	2.32 (1.03)	0.14-3.56

**Table:** (c) Comfortable sustained phonation

Protocol parameter	18–39 y (*n* = 76)		40–59 y (*n* = 70)		60–87 y (*n* = 60)	
Comfortable sustained phonation	Mean (SD)	Min–Max	Mean (SD)	Min–Max	Mean (SD)	Min–Max
Female	*n* = 41		*n* = 35		*n* = 30	
Maximum SPL (dB)	97.28 (5.03)	89.44-107.59	98.23 (5.52)	87.99–109.99	98.68 (5.04)	90.67-111.03
Minimum SPL (dB)	92.78 (5.11)	83.45-103.22	93.31 (5.37)	84.36-102.84	93.09 (4.92)	84.21-104.69
Mean SPL (dB)	95.04 (5.02)	86.13-105.60	95.84 (5.32)	86.13-105.68	95.95 (4.97)	88.56-108.00
SPL range (dB)	4.51 (1.37)	1.79-8.23	4.92 (2.31)	2.05-13.53	5.69 (2.28)	3.00-13.17
Mean pitch (Hz)	227.80 (22.52)	173.99-255.92	226.20 (27.58)	166.46-259.64	206.68 (29.16)	141.07-255.75
Phonation time (s)	5.00 (0.00)	6.83–8.58	5.00 (0.00)	6.95-8.92	5.00 (0.00)	6.51–7.52
Peak expiratory airflow (L/s)	0.14 (0.06)	0.03-0.34	0.17 (0.07)	0.05-0.40	0.15 (0.07)	0.01-0.39
Mean expiratory airflow (L/s)	0.11 (0.05)	0.02-0.28	0.12 (0.05)	0.03-0.29	0.11 (0.05)	0.0067-0.20
Expiratory volume (L)	0.60 (0.27)	0.12-1.42	0.64 (0.29)	0.17-1.49	0.56 (0.27)	0.03-1.03

Male	*n* = 35		*n* = 35		*n* = 30	
Maximum SPL (dB)	97.79 (4.45)	86.28-108.37	100.57 (5.83)	88.60-114.89	99.14 (5.33)	87.06-108.31
Minimum SPL (dB)	92.50 (5.61)	80.82–103.90	95.35 (8.37)	64.22-112.35	92.75 (9.89)	57.36-104.37
Mean SPL (dB)	95.49 (4.51)	83.46-106.15	98.19 (6.10)	86.50-113.95	96.00 (7.12)	77.93-106.19
SPL range (dB)	5.32 (2.48)	2.40-13.13	5.22 (6.53)	2.39-42.28	6.50 (6.27)	2.19-36.95
Mean pitch (Hz)	130.33 (18.01)	101.39-169.45	147.87 (33.02)	102.70-225.23	155.83 (45.89)	82.70-261.63
Phonation time (s)	5.00 (0.00)	6.83–7.68	5.00 (0.00)	7.01–8.15	5.00 (0.00)	6.35–7.45
Peak expiratory airflow (L/s)	0.227 (0.08)	0.05-0.38	0.33 (0.57)	0.03-3.57	0.40 (0.94)	0.07-5.37
Mean expiratory airflow (L/s)	0.16 (0.07)	0.03-0.31	0.18 (0.09)	0.02-0.41	0.16 (0.08)	0.04-0.35
Expiratory volume (L)	0.83 (0.35)	0.17-1.58	0.92 (0.45)	0.13-2.05	0.82 (0.41)	0.21-1.76

**Table:** (d) Variation in sound pressure level

Protocol parameter	18–39 y (*n* = 76)		40–59 y (*n* = 70)		60–87 y (*n* = 60)	
Variation in sound pressure level	Mean (SD)	Min–Max	Mean (SD)	Min–Max	Mean (SD)	Min–Max
Female	*n* = 41		*n* = 35		*n* = 30	
Maximum SPL (dB)	106.01 (4.35)	96.90-113.21	108.14 (4.35)	97.36-114.27	107.72 (3.84)	99.12-113.74
Minimum SPL (dB)	83.20 (6.92)	63.06-91.81	84.97 (6.83)	64.70-93.59	85.83 (10.26)	63.42-100.04
Mean SPL (dB)	95.12 (4.27)	87.02-103.85	97.54 (3.67)	90.28-104.31	98.00 (3.85)	88.21-104.32
SPL range (dB)	22.72 (6.62)	11.92-41.28	22.98 (8.62)	9.90-46.54	21.77 (9.82)	6.93-47.72
Mean pitch (Hz)	221.96 (19.28)	181.99-249.51	211.76 (16.35)	182.79-241.59	202.92 (20.82)	163.04-249.19
Pitch range (Hz)	90.97 (40.59)	31.06-173.36	106.76 (35.01)	47.14-163.35	91.15 (36.12)	27.51-179.06
Target airflow (L/s)	0.11 (0.05)	0.01-0.22	0.14 (0.05)	0.03-0.30	0.10 (0.05)	0.0067-0.24

Male	*n* = 35		*n* = 35		*n* = 30	
Maximum SPL (dB)	107.38 (4.58)	90.17-114.47	107.22 (6.99)	75.85-115.25	108.80 (4.08)	100.33-114.17
Minimum SPL (dB)	86.87 (5.63)	62.35-96.93	85.05 (11.60)	61.65-99.02	90.73 (5.92)	75.48-99.41
Mean SPL (dB)	97.39 (5.67)	76.41-107.60	99.11 (5.42)	84.40-109.22	100.61 (4.86)	88.34-108.12
SPL range (dB)	20.64 (6.28)	10.61-45.55	23.09 (10.26)	9.96-43.86	18.06 (4.95)	6.85-32.87
Mean pitch (Hz)	139.04 (21.71)	110.99-191.94	149.15 (23.64)	105.36-198.93	162.40 (29.01)	114.39-211.05
Pitch range (Hz)	61.00 (32.26)	10.76-134.33	80.08 (36.12)	15.15-168.20	82.59 (35.71)	25.07-195.33
Target airflow (L/s)	0.23 (0.10)	0.03-0.43	0.26 (0.11)	0.07-0.53	0.22 (0.11)	0.02-0.43

**Table:** (e) Voicing efficiency

Protocol parameter	18–39 y (*n* = 76)		40–59 y (*n* = 70)		60–87 y (*n* = 60)	
Voicing efficiency	Mean (SD)	Min–Max	Mean (SD)	Min–Max	Mean (SD)	Min–Max
Female	*n* = 41		*n* = 35		*n* = 30	
Maximum SPL (dB)	98.13 (5.0)	89.85-109.47	99.92 (5.66)	90.71-111.52	99.84 (5.16)	90.31-111.37
Mean SPL (dB)	94.70 (4.53)	87.70-104.52	96.67 (5.37)	88.17-107.73	96.43 (5.07)	87.26-107.75
Mean SPL during voicing (dB)	94.87 (4.48)	87.70-104.47	96.69 (5.32)	88.17-107.73	96.46 (5.06)	87.29-107.74
Mean pitch (Hz)	217.44 (24.14)	162.73-262.45	214.83 (28.45)	145.61–252.34	194.00 (25.84)	154.93-244.84
Pitch range (Hz)	32.95 (25.70)	8.38-98.25	33.26 (32.53)	10.31-154.53	34.54 (30.11)	9.19-134.60
Expiratory airflow duration (s)	0.91 (0.30)	0.39-1.85	1.08 (0.24)	0.55-1.67	1.19 (0.30)	0.69-1.79
Peak air pressure (cm H_2_O)	9.47 (3.57)	4.95-22.81	10.61 (3.45)	6.03-20.93	10.87 (2.87)	6.49-17.83
Mean peak air pressure (cm H_2_O)	7.67 (2.33)	3.81-14.92	8.39 (2.56)	4.95-15.53	8.88 (2.43)	5.10-15.05
Peak expiratory airflow (L/s)	0.15 (0.09)	0.03-0.51	0.17 (0.06)	0.06-0.34	0.15 (0.08)	0.01-0.32
Target airflow (L/s)	0.09 (0.04)	0.01-0.26	0.12 (0.05)	0.04-0.22	0.10 (0.06)	0.0033-0.24
Expiratory volume (L)	0.08 (0.05)	0.01-2.26	0.13 (0.06)	0.03-0.30	0.13 (0.10)	0.0033-0.36
Mean airflow during voicing (L/s)	0.09 (0.04)	0.01-0.27	0.11 (0.04)	0.03-0.21	0.09 (0.06)	0.0033-0.24
Aerodynamic power (W)	0.08 (0.06)	0.01-0.37	0.10 (0.05)	0.03-0.22	0.08 (0.06)	0.0047-0.23
Aerodynamic resistance (cm H_2_O/L/Sn)	130.38 (142.60)	36.75-756.10	87.83 (52.89)	25.75-229.71	380.36 (692.39)	35.86-3167.15
Acoustic ohms (dyne s cm^5^)	132.96 (145.43)	37.48-771.06	89.53 (53.71)	26.26-234.26	389.02 (705.74)	36.56-3229.80
Aerodynamic efficiency (dyne s cm^5^)	22325.21 (59863.48)	299–385353	16485.89 (20175.95)	882.02-84885.30	49606.61 (112786.19)	1198.12-533250.63

Male	*n* = 35		*n* = 35		*n* = 30	
Maximum SPL (dB)	97.85 (4.40)	85.55-106.57	100.85 (5.88)	90.23-111.63	100.18 (5.11)	84.01-110.85
Mean SPL (dB)	94.73 (4.57)	81.82-103.25	97.63 (6.83)	78.23-109.04	96.82 (5.15)	80.36-105.87
Mean SPL during voicing (dB)	94.82 (4.50)	81.82-103.25	97.85 (6.55)	82.81-109.04	99.53 (17.10)	80.38-186.33
Mean pitch (Hz)	123.03 (14.69)	98.04-154.05	137.22 (24.85)	94.77-201.48	149.709 (28.96)	103.40–215.93
Pitch range (Hz)	14.63 (15.14)	4.86-93.16	16.01 (9.89)	4.65-60.54	21.58 (12.68)	9.29-62.20
Expiratory airflow duration (Sn)	0.83 (0.32)	0.38-1.65	1.05 (0.31)	0.47-1.66	1.05 (0.30)	0.60-1.74
Peak air pressure (cm H_2_O)	10.81 (5.00)	4.85-32.68	11.85 (5.34)	4.46-23.22	14.64 (6.06)	7.11-33.90
Mean peak air pressure (cm H_2_O)	8.71 (4.01)	3.82-26.04	9.25 (3.96)	3.58-20.06	10.98 (3.90)	5.13-17.59
Peak expiratory airflow (L/s)	0.38 (0.27)	0.03-1.03	0.38 (0.18)	0.08-0.86	0.31 (0.17)	0.0067-0.70
Target airflow (L/s)	0.21 (0.11)	0.02-0.46	0.23 (0.10)	0.05-0.53	0.19 (0.11)	0.001-0.44
Expiratory volume (L)	0.17 (0.11)	0.10-0.59	0.24 (0.14)	0.04-0.57	0.20 (0.14)	0.001-0.59
Mean airflow during voicing (L/s)	0.20 (0.11)	0.02-0.46	0.22 (0.10)	0.05-0.51	0.18 (0.11)	0.001-0.43
Aerodynamic power (W)	0.20 (0.16)	0.01-0.59	0.22 (0.15)	0.03-0.59	0.21 (0.15)	0.002-0.59
Aerodynamic resistance (cm H_2_O/L/Sn)	100.49 (236.97)	20.46-1386.54	48.61 (30.97)	13.33-173.04	174.85 (420.55)	12.09-2285.61
Acoustic ohms (dyne s cm^5^)	102.87 (241.53)	22.64-1413.97	49.38 (31.66)	13.59-176.47	178.31 (428.87)	12.34-2330.83
Aerodynamic efficiency (ppm)	11102.94 (38165.33)	229.66-228739.41	8507.71 (8648.68)	105.53-35164.99	9594.30 (14383.53)	1259.95-78035.27

## Data Availability

The datasets used and/or analyzed during the current study are available from the corresponding author on reasonable request.
